# Highly Porous Carbon Flakes Derived from Cellulose and Nickel Phosphide Heterostructure towards Efficient Electrocatalysis of Oxygen Evolution Reaction

**DOI:** 10.3390/molecules29020352

**Published:** 2024-01-10

**Authors:** Ewa Mijowska, Karolina Pietrusewicz, Klaudia Maślana

**Affiliations:** 1 Department of Nanomaterials Physicochemistry, Faculty of Chemical Technology and Engineering, West Pomeranian University of Technology, Piastow Ave. 45, 70-311 Szczecin, Poland; 2 Center for Advanced Materials and Manufacturing Process Engineering (CAMMPE), West Pomeranian University of Technology, 70-310 Szczecin, Poland

**Keywords:** cellulose, oxygen evolution reaction, nickel phosphides, electrochemistry

## Abstract

This study delves into the pressing challenges of climate change and the escalating carbon dioxide (CO_2_) emissions by exploring hydrogen technology as a sustainable alternative. In particular, there is focus on nickel phosphide-based electrocatalysts, known for their promising performance in hydrogen evolution reactions (HERs) and oxygen evolution reactions (OERs). Therefore, here we have designed a facile strategy to deliver highly porous carbon flakes derived from cellulose fibers via carbonization at 850 °C, yielding highly porous structures and outstanding specific surface area (SSAcel_carb_850_act = 3164 m^2^/g) after activation. As-fabricated carbon was utilized as a support for Ni_12_P_5_ with an optimized mass ratio. Electrochemical testing revealed that the composite of Ni_12_P_5_ and carbon flakes with a ratio of 100:1, respectively, exhibited the most favorable kinetics for the oxygen evolution reaction (OER). Importantly, the durability tests of this sample demonstrated the most stable behavior and lowest potential change under high current density among the studied samples, making it a promising candidate in practical applications. Moreover, the analysis of electrocatalysts after an OER does not show any changes, indicating that the sample does not undergo undesired intermediate reactions and that unwanted products are not released, explaining its stable behavior. This provides a straightforward approach for creating a cellulose-derived composite with enhanced electroactivity and durability.

## 1. Introduction

Climate change, global warming, and carbon dioxide (CO_2_) emissions have been the most frequently heard keywords in recent years [1]. Despite a slowdown in the process, data reveal a continuous and steady increase in CO_2_ emissions into the atmosphere [1]. In 2022, it increased by 1%, which corresponded to 37.5 Gt of CO_2_ being emitted into the atmosphere [1]. The gas is mainly created by burning fossil fuels, especially their petroleum derivatives, but their reserves are slowly depleting [2,3]. In addition, the continued rise in prices and the serious threat to the environment are pushing scientists to search for new methods of obtaining emission-free, as well as less costly, renewable green fuels [4,5]. This search brings us to hydrogen technology. 

Hydrogen, also known as the fuel of the future, is proving to be a good successor to existing energy sources [6]. The production of hydrogen energy does not require combustion, meaning no greenhouse gases are produced, and the only byproduct is water [7,8,9,10]. In addition, it is easily available and can be produced from renewable resources such as biomass or water [11,12,13,14]. However, the cost of hydrogen production has been a significant barrier to its widespread adoption. Depending on the hydrogen source, three main colors of hydrogen can be distinguished: (i) grey hydrogen (e.g., from direct steam methane reforming (SMR), during which CO_2_ is emitted as byproduct), (ii) blue hydrogen (from SMR, during which CO_2_ is captured), and (iii) green hydrogen (from electrolysis with utilization of renewable energy sources) [15,16]. Green hydrogen, despite the fact that it is considered the most environmentally friendly, currently accounts for only ~4% of the world’s total energy use. This figure encompasses various industrial applications, such as fertilizers, metallurgy, and chemicals, where hydrogen is utilized [17,18]. The cost of H_2_ production has been a significant barrier to its widespread adoption. Grey hydrogen has traditionally been the cheapest, but there is a growing focus on reducing the cost of green hydrogen [15]. As the hydrogen industry grows, the overall costs are expected to decrease. Rising demand, technological progress, and efficient production processes will further contribute to cost reductions. To create the hydrogen economy, significant infrastructure investment in production plants, distribution networks, and storage is required [17]. Each of these three aspects constitutes a separate aspect of the development of the hydrogen economy. When considering production plants, it can be noticed that continuous research on hydrogen production technologies is fostering innovation and efficiency gains, resulting in cost reductions. Decreasing costs of renewable energy, a crucial factor in green hydrogen production, enhance overall competitiveness. Currently, the electrochemical reaction of water splitting is the most investigated method for green hydrogen production [19], in which two simultaneous reactions can be distinguished: the hydrogen evolution reaction (HER) and the oxygen evolution reaction (OER) [11,20,21]. In these processes, the employment of appropriate electrocatalysts is essential. However, the currently utilized catalysts, crafted from noble materials, such as platinum, ruthenium, and iridium, proved to be excessively costly and challenging to obtain [22]. For this reason, the main goal is to continue the search for equally good, stable, and sustainable substitutes. Currently, the attention of researchers is focused on d-block non-precious metals due to their low production cost and widespread occurrence in nature [23,24,25]. Therefore, they have been recognized as promising alternatives as catalysts for hydrogen/oxygen evolution reactions [26,27,28]. Among them, in recent years, electrocatalysts based on nickel phosphides have gained focus, showing promising performance for HERs and OERs [22,29]. Nickel phosphides exhibit notable catalytic activity, kinetics, and cost-effectiveness as catalysts in both HERs and OERs, making them promising candidates for efficient electrocatalysts. Unlike other transition metal phosphides, nickel phosphides are characterized by their exceptional combination of high catalytic activity, electronic conductivity, tunability in terms of composition and structure, and favorable stability under conditions relevant to water electrolysis [30,31]. Additionally, the abundance and cost-effectiveness of nickel as a transition metal further enhances the appeal of nickel phosphides, making them stand out as promising electrocatalysts for hydrogen production and other applications in the realm of sustainable energy technologies [32]. Despite their good performance, they exhibit some limitations, e.g., degradation in long-term stability hinders their commercial application. Therefore, many attempts have been made to modify the surface of nickel phosphides, leading to the improvement of electrochemical properties. The deposition of nanoparticles onto the molecular matrix serves as a strategic approach to enhance the properties of HERs and OERs [26,28]. The incorporation of nanoparticles onto a supportive matrix leads to an increment of electrocatalysts’ active surface, providing more sites for electrochemical reactions [28,31]. Additionally, the support offers structural stability and avoids the aggregation of nanoparticles, ensuring prolonged durability and sustained electrocatalytic performance [29]. Several materials can serve as platforms for this purpose, such as carbon nanotubes, graphene, metal oxides, metal–organic frameworks, and others [29,33,34]. Cellulose is an interesting approach due to its huge abundance in natural resources [35]. Moreover, cellulose has numerous active functional groups in its nature which allow easy functionalization with other chemical compounds [36]. However, cellulose shows low conductivity and, to boost it, the carbonization/activation process can be applied. This process also enables the substitution of certain carbon atoms with heteroatoms (e.g., nitrogen, boron, phosphorus, sulfur) and promotes specific surface areas affecting the catalytic performance. 

This study is focused on the carbonization process of cellulose to provide highly porous flake-like support for nickel phosphide (Ni_12_P_5_), which can serve as an efficient and robust electrocatalyst in the oxygen evolution reaction during water decomposition. Systematic study of the electrocatalytic performance of the composites with different mass ratios of Ni_12_P_5_ to carbon flakes allowed us to reveal the most promising composition in terms of practical applications. The microscopic and structural nature of the samples has been evaluated by TEM, Raman spectroscopy, XRD, and N_2_ adsorption/desorption isotherms. Tafel slope, EIS, and chronopotential tests for long-term stability revealed that sample of Ni_12_P_5__cellulose_100:1 is the most promising. Therefore, this catalyst after the OER was investigated and it was proved that no phase changes occurred, which meant that it did not undergo undesired intermediate reactions and that unwanted products were not released, which highlighted its stable behavior. Finally, the reaction mechanism is proposed. 

## 2. Results 

**Figure 1:**
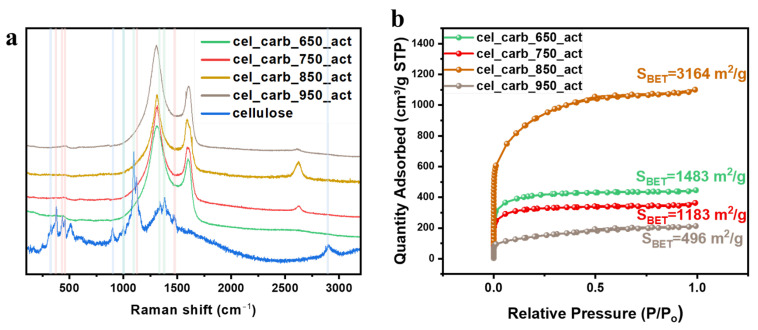
(**a**) Raman spectra; (**b**) N_2_ adsorption/desorption isotherms of carbonized cellulose with different temperatures after the activation process.

SEM images of cellulose fibers before and after the ball milling process are presented in Figure S1. Figure S2 demonstrates the morphology of samples after the carbonization process. The chemical structures and thermal stability are shown in Figure S3. Raman analysis is usually performed to investigate the structure of pristine cellulose after the activation process. Many secondary and tertiary structures can be formed from cellulose molecules. This is caused by variations in intra- and intermolecular hydrogen bonds, as well as chain polarity and parallel versus antiparallel chains. Each of these aspects has an impact on the crystal parameters of the various allomorphs (crystalline phases), which, in turn, determines the accessibility of the structure as a whole [37]. Therefore, cellulose can exhibit at least six polymorphs—cellulose I, cellulose II, cellulose III, cellulose III_II_, cellulose IV_I_, and cellulose IV_II_. Raman spectroscopy can be used to study and determine the type of cellulose present in the sample. Therefore, in Figure 1a, the signals assigned to different types of cellulose were highlighted (cellulose I—red, cellulose II—green, and cellulose III—blue) [37]. The used cellulose was a mixture of different types of cellulose crystalline structures. However, after carbonization and activation, the peaks typical for cellulose completely disappeared, and only a small signal around 460 cm^−1^ was detected. Two strong peaks associated with carbon dominate the spectrum—D band at ~1308 cm^−1^ which corresponds to disordered planes/defects and the G peak at ~1603 cm^−1^ attributed to graphitic planes [38]. The crystallinity of graphitic carbon is estimated based on the ratio of D band to G band intensity (I_D_/I_G_). Due to the presence of edge defects, the I_D_/I_G_ ratio tends to increase with decreasing lateral size [39,40]. Therefore, values of I_D_/I_G_ ratios were calculated and are presented in Table 1. The I_D_/I_G_ ratio for the sample of cel_carb_850_act is the lowest, indicating that it possesses the least structural defects. However, overall, the intensity of the D mode in all spectra is high, which means that all the fabricated samples are composed mainly of amorphous carbons. Moreover, after the activation process, the 2D mode at ~2627 cm^−1^ appears for two samples: cel_carb_750_act and cel_carb_850_act. However, it is much better pronounced in cel_carb_850_act. The appearance of the 2D signal is usually associated with the carbon samples composed of graphene layers [41]. 

N_2_ adsorption/desorption analysis was performed to determine specific surface area, as well as micropore volume and area (Figure 1b). The collected isotherms of all samples represent type I isotherms, indicating the micropore structure of samples [42,43]. The plot obtained for sample cel_carb_850_act shows a significantly higher amount of N_2_ adsorbed, resulting in a higher specific surface area (3164 m^2^/g). The results calculated from the N_2_ adsorption/desorption analysis are summarized in Table 1.

**Figure 2:**
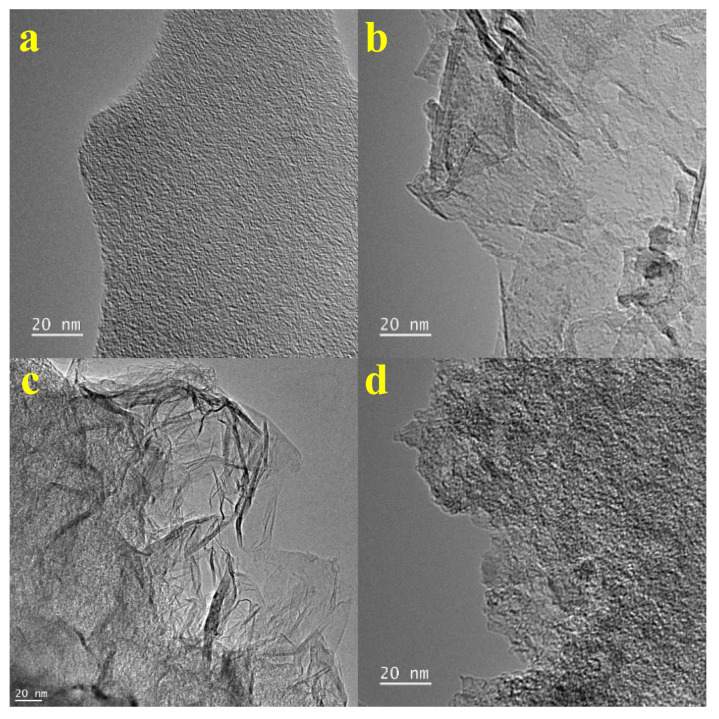
TEM images of cellulose carbonized at different temperatures after the activation process: (**a**) 650 °C, (**b**) 750 °C, (**c**) 850 °C, (**d**) 950 °C.

TEM images of carbonized cellulose at different temperatures after the activation process provide valuable insights into the structural evolution of the material (Figure 2). Both samples of cel_carb650_act and cel_carb950_act (Figure 2a,d) exhibit similar material structures with a well-defined network of interconnected pores. Despite the higher carbonization temperature of sample cel_carb950_act, the similarity to the cel_carb650_act suggests that the activation process might have led to the preservation of pore structures. In contrast, samples cel_carb750_act and cel_carb850_act (Figure 2b,c) reveal a thin-layered structure, suggesting the formation of carbon layers during the activation process. However, cel_carb750_act exhibits more defects compared to the sample carbonized at 850 °C, indicating a less controlled or more dynamic carbonization process. The increased defects might contribute to a more irregular and less ordered structure. The sample cel_carb850_act appears the most ordered, but the degree of graphitization is still low and resembles a typical amorphous structure which was also proved by Raman spectroscopy (Figure 1).

**Figure 3:**
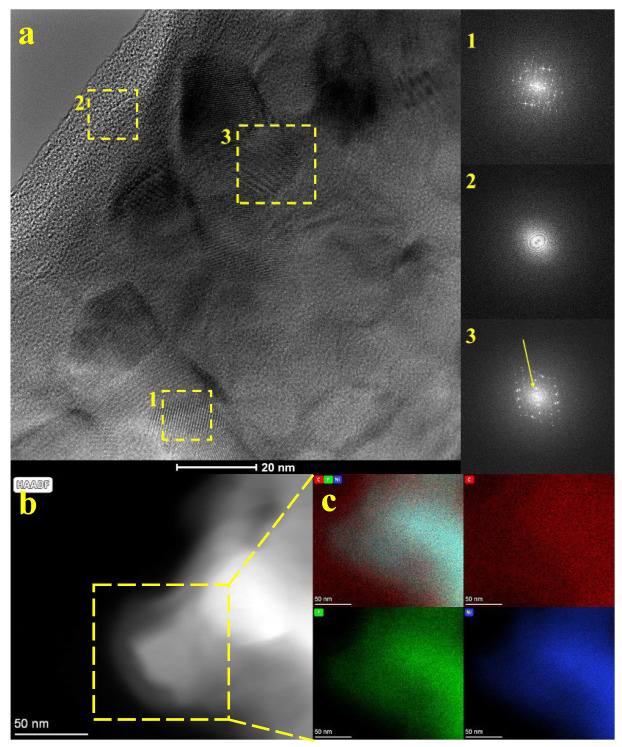
(**a**) TEM analysis of Ni_12_P_5__cel_100:1 with corresponding FFT images, (**b**) STEM image with (**c**) corresponding elemental EDS mapping of signals overlayered from all elements: carbon, phosphorus, and nickel.

Subsequently, the morphology of cellulose and nickel phosphide composites with varied mass ratios of components (Ni_12_P_5_:cellulose) was evaluated by TEM (Figure 3 and Figure S4). With lower Ni_12_P_5_ content, cellulose flakes dominate but dispersed nickel phosphide nanoparticles are detected. Intermediate ratios reveal a more intricate composite structure with increased nickel phosphide forming clusters. Higher ratios showcase a densely packed or agglomerated arrangement of nickel phosphide, overshadowing cellulose fibers. The TEM images demonstrate significant aggregation in the nanoparticle distributions. The micrographs were analyzed to determine the average particle size. The average size of individual nanoparticles was determined based on measurements of 50 individual particles and was ~22 nm, 64 nm, 3 nm, 14 nm, and 47 nm for Ni_12_P_5__cel_100:1, Ni_12_P_5__cel_10:1, Ni_12_P_5__cel_1:1, Ni_12_P_5__cel_1:10, and Ni_12_P_5__cel_1:100, respectively. Detailed TEM analysis was performed for the sample with the highest Ni_12_P_5_ content in the sample and is presented in Figure 3a, along with fast Fourier transform (FFT) patterns taken from regions marked in the boxes. Ni_12_P_5_ nanoparticles are deposited on the carbon layer, albeit exclusively in the inner region because the amorphous carbon edges of the flakes are clearly detected, which is proved by FFT (the ring patterns (box no. 2)). However, the FFT of nickel phosphide (box no. 1) displays a single-crystalline phase with d-spacing of 2.21 Å, which corresponds to the (111) plane of Ni_2_P (ICDD no. 04-008-0034). Moreover, in the area marked with box no. 3, the FFT patterns revealed a single-crystalline phase of nickel phosphide together with rings related to amorphous carbon (marked with a yellow arrow), indicating its deposition onto carbon. Additionally, the sample was examined under AFM microscopy, and the results are presented in Figure S5. It was shown that the average particle thickness (height) of Ni_12_P_5__cellulose_100:1 was 8.2 nm. Figure 3b shows a STEM image of the sample with the corresponding area of EDX mapping marked with a yellow square. Figure 3c shows the elemental mapping of Ni, P, and C. The EDX mapping images show a uniform distribution of P and Ni atoms throughout the sample. This even distribution suggests that these elements are well-distributed, indicating a homogeneous composition. The images further reveal the presence of a carbon layer surrounding Ni_12_P_5_ particles. This suggests a specific structural arrangement where Ni_12_P_5_ nanoparticles are distributed on a carbon layer mostly on its inner surface, leaving the carbon platform edges uncoated. The carbon layer serves as a protective barrier preventing it from agglomeration and may also influence the sample’s properties, potentially affecting its reactivity or stability. Additionally, an enhanced carbon signal is observed in the EDX mapping images, which is attributed to the presence of a carbon film on the TEM grid.

**Figure 4:**
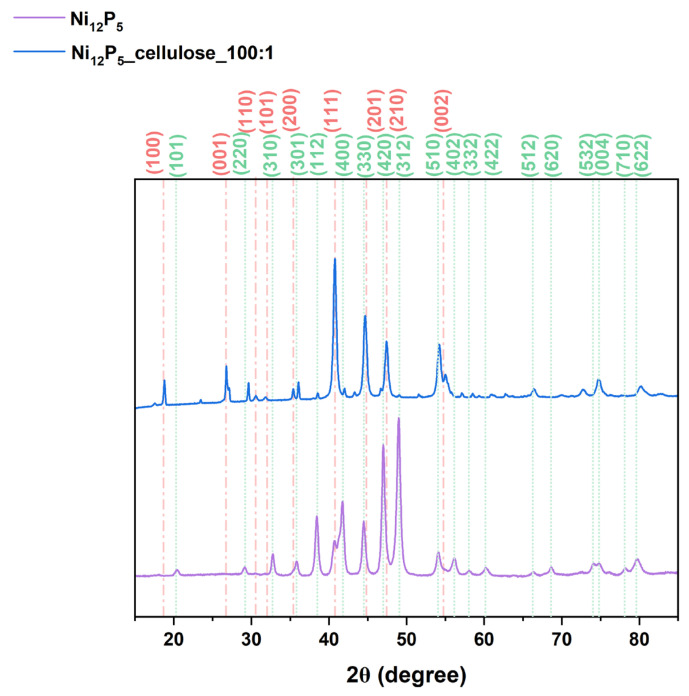
XRD patterns of Ni_12_P_5_ and Ni_12_P_5__cellulose_100:1. The red and green lines are assigned to Ni_2_P and Ni_12_P_5_ phases, respectively.

The crystallographic structure was examined using X-ray diffraction analysis and the composition of the resulting composites was confirmed (Figure 4 and Figure S6). According to the XRD patterns, pristine Ni_12_P_5_ exhibits a single-phase composition of Ni_12_P_5_, which consists of signals marked in the red line in Figure 4, and they correspond to 2θ = 32.71°, 38.42°, 41.74°, 46.96°, 48.96°, and 60.14° (JCPDD no. 00-022-1190). Interestingly, after the deposition of Ni_12_P_5_ on a carbon support, additional signals corresponding to Ni_2_P also occur (marked with a green line). The signals with the highest intensities from Ni_2_P are 2θ = 17.49°, 40.92°, 44.82°, and 54.75° (ICDD no. 04-001-9848). This can be due to the interaction of phosphide with carbon, which facilitates a partial carboreduction process [44,45] of Ni_12_P_5_ to Ni_2_P. Additionally, the intense peaks of nickel phosphides with narrow full width at half maximum (FWHM) indicate the crystalline structure of compounds, whereas with increasing carbon content in the sample, especially in the sample of Ni_12_P_5__cellulose_1:100, clear signals from carbon at 2θ = 26.5° and 44.59° are detected (ICDD no. 00-023-0064). 

Electrochemical performance was investigated by several methods. Kinetics and dynamics versus the stability/durability of the electrocatalysts were tested. However, although stability is the most important parameter worth analyzing, tests such as LSV, EIS, and Tafel slope allow for in-depth knowledge of the course of the reaction. The analysis of such data allows for precise planning of further modifications for more effective electrocatalysts.

**Figure 5:**
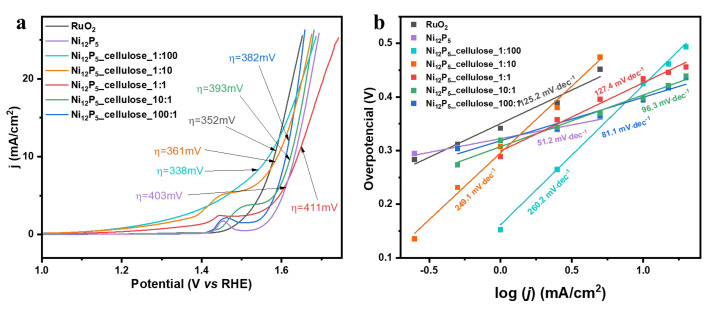
(**a**) LSV and (**b**) Tafel plots of RuO_2_, Ni_12_P_5_, and Ni_12_P_5__cellulose composites.

LSV was used to determine the overpotential value with a commercial RuO_2_ catalyst used as a reference in the reaction. In Figure 5a, it is demonstrated that the Ni_12_P_5__cel_1:100 sample exhibited the smallest overpotential at 10 mA cm^−2^ current density (η = 338 mV). In general, the smaller the overpotential value, the less external energy must be supplied to the electrocatalysts to start the OER process, which is advantageous from an economic point of view. Ni_12_P_5__cel_100:1, Ni_12_P_5__cel_10:1, Ni_12_P_5__cel_1:1, and Ni_12_P_5__cel_1:10 display overpotentials of 382 mV, 393 mV, 411 mV, and 361 mV, respectively, when the current density is 10 mA cm^−2^. The Ni_12_P_5__cel_1:100 electrocatalyst outperforms the commercial RuO_2_. The overpotential value for Ni_12_P_5__cel_1:100 decreased by about 14 mV concerning RuO_2_. The lowest overpotential was obtained for samples, where the ratio of Ni_12_P_5_ and cellulose is the smallest, indicating the majority of cellulose in the sample. It is assumed that this is due to the uniform deposition of Ni_12_P_5_ nanoparticles on the carbon sample which results in exposure of the active sites of the sample, which is in good correlation with the TEM results (Figure 3). 

In addition, the Tafel slope is also an essential parameter for electrocatalytic activity which gives insights into the electrocatalytic activity of a material in electrochemical reactions. Generally, the lower the Tafel slope is, the more efficient the electrocatalyst is. Figure 5b illustrates that, among the obtained composites, Ni_12_P_5__cel_100:1 exhibits the lowest Tafel slope of 81.1 mV·dec^−1^, while the slopes of Ni_12_P_5__cel_10:1, Ni_12_P_5__cel_1:1, Ni_12_P_5__cel_1:10, and Ni_12_P_5__cel_1:100 are 96.3 mV·dec^−1^, 127.4 mV·dec^−1^, 249.1 mV·dec^−1^, and 260·2 mV·dec^−1^_,_ respectively. Contrary to overpotential results, here, with increasing Ni_12_P_5_ content in the sample, the Tafel slope value is decreasing, indicating the change in the rate-determining step of the process followed by the reaction kinetics improvement. 

**Figure 6:**
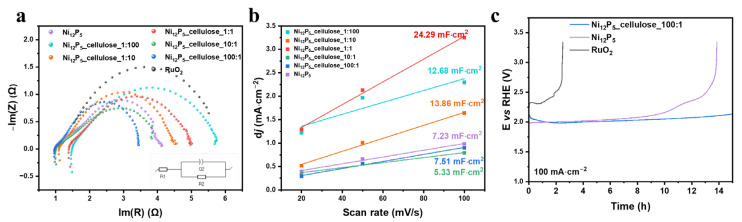
(**a**) EIS results with an insert of R1 + Q2/R2 equivalent circuit used as a fitting model, (**b**) current density vs. scan rate plot, and (**c**) stability results performed at 100 mA/cm^2^ for 15 h.

The examination of charge transfer at the interface between electrocatalysts and electrolytes serves as a crucial determinant in assessing the OER. Electrochemical Impedance Spectroscopy (EIS) reveals the dynamics of the charge transfer, indicating the underlying reaction mechanism. Consequently, EIS measurements were conducted to offer deeper insights into the electrode kinetics of the acquired materials. In Figure 6a, Nyquist plots and fitting outcomes to the R1 + Q2/R2 equivalent circuit are depicted, employing an appropriate model (refer to the insert in Figure 6a). This model encompasses a solution resistance (R1), charge transfer, and/or mass transfer resistances (R2), with Q2 being associated with the time-constant dispersion of the measured dielectric constant [Fs^(α−1)^] at the catalyst/electrolyte interface. The resistances acquired from the samples are listed in Table 2, indicating that the rate-determining step aligns with R2. This parameter is associated with electrochemical charge transfer resistance and/or redox reactions. The magnitude of the R2 value is depicted by the semicircle diameter, with a larger diameter indicating increased resistance. The data revealed that most synthesized materials possess lower R2 values compared to RuO_2_. Ni_12_P_5__cellulose_1:100 does not follow this trend, which can be due to it presenting the smallest content of phosphides, which are responsible for boosting the conductivity of other samples. The most promising results were obtained for the Ni_12_P_5__cellulose_100:1 sample, where the R2 value decreased by over 32% compared to RuO_2_. Moreover, the R2 value for this sample was lower than pure Ni_2_P, which suggests the synergistic effect of cellulose and Ni_2_P lowering the energy barrier of the OER, thereby accelerating the reaction kinetics. This result is in line with the value of the Tafel slope of Ni_12_P_5__cellulose_100:1.

To estimate the electrochemical active surface area (*ECSA*), the double-layer capacitance (*C_dl_*) was calculated via the CV curves at the non-Faradaic region. The determination of ECSA was carried out by evaluating its interaction with the double-layer capacitance (*C_dl_*). Moreover, from the *C_dl_*, the *ECSA* of the electrocatalysts can be calculated using the following equation:

$$ECSA=\frac{c_{dl}}{c_{dl\left(s\right)}}$$

where *C_dl_*_(*S*)_ is the *C_dl_* for an electrode substrate. Hence, we have chosen an average of 0.04 mF·cm^−2^ as a theoretical *C_dl_* of the flat graphitic surface. The calculated *ECSA* values were 180.75 cm^2^, 187.75 cm^2^, 133.25 cm^2^, 607.25 cm^2^, 346.50 cm^2^, and 317.00 cm^2^ for Ni_12_P_5_, Ni_12_P_5__cel_100:1, Ni_12_P_5__cel_10:1, Ni_12_P_5__cel_1:1, Ni_12_P_5__cel_1:10, and Ni_12_P_5__cel_1:100. Almost all composites exhibit higher ECSA values compared to pure Ni_12_P_5_. It is interesting to note is that the sample Ni_12_P_5__cel_1:1, which has an equal ratio of carbon to nickel phosphide, exhibits the highest ECSA value. The 1:1 ratio might provide an optimal balance between the conductive carbon matrix and the catalytically active nickel phosphide. However, the samples composed of majority carbon (ratio 1:10 and 1:100) possess lower *ECSA* values compared to 1:1. This phenomenon could be due to the fact that an excess of carbon does not contribute significantly to the electrochemical surface area. Too much carbon might lead to a dilution effect, reducing the density of active sites for electrochemical reactions.

The stability test was performed for the samples Ni_12_P_5_, Ni_12_P_5__cellulose_100:1, and RuO_2_. The tests were performed with a high current density of 100 mA·cm2 for 15 h. Figure 6c shows unfavorable behavior in RuO_2_, displaying unstable results with high starting potential and sudden potential growth after 2.5 h, concluding the test after approximately 3 h. In contrast, Ni_12_P_5__cellulose_100:1 demonstrates the most promising stability properties, maintaining the lowest potential value through the full 15 h. At 2 h after the start of the test, the starting potential decreased by approximately −7%, suggesting activation of the electrocatalyst after 2 h. The test ended with a slight increase in potential of about +8.5%. However, Ni_12_P_5_ exhibited less stability, with a sharp potential increase after 8 h and complete degradation after 13 h, indicating a significant loss of stability in a relatively short period. Additionally, this sample showed the lowest Tafel slope values of all the samples. Therefore, from a practical application point, this composition is the most promising and it should undergo, in further study, deeper electrochemical investigations such as LSV or stability tests at much higher current densities or for prolonged times (e.g., 1000 h).

During the study on the comparison of our results with the existing literature, it was clearly noticed that it contributes to filling the knowledge gap in the field of cellulose-derived substrates for electroactive nickel phosphides in the OER. However, there are some interesting reports dealing with other biomass-derived and synthetic carbon matrixes. For example, Xiong et al. [46] synthesized Ni*_7_*P*_3_* particles on a lignin-derived carbon platform. The obtained electrocatalyst was deposited on carbon paper and tested towards the OER in 1.0 M KOH. They obtained an overpotential value equal to 350 mV and a Tafel slope of 176 mV/dec. On the other hand, Shanmugam et al. [47] tested NiP embedded in amorphous carbon towards the OER in 1 M KOH using a glassy carbon electrode. As they have shown, prepared electrocatalysts revealed the overpotential value of 380 mV at 10 mA·cm*^−2^* and Tafel slope equal to 106.7 mV/dec. In contrast, Aziz et al. [48] coupled Ni*_12_*P*_5_* with mildly oxidized multiwall carbon nanotubes, which resulted in a 280 mV overpotential value and a 62 mV/dec Tafel slope in 1M KOH electrolyte. However, the use of synthetic carbon nanotubes excludes their use as sustainable electrocatalysts, which is a significant aspect of our research. Additionally, our method stands out for its simplicity and offers the potential for utilizing cellulose-based waste materials to enhance the efficiency of the catalyst. This environmentally friendly approach not only simplifies the production process but also contributes to the sustainable utilization of resources, making our catalyst an attractive option in the pursuit of efficient and eco-friendly electrocatalyst solutions.

### 2.1. XPS Analysis

**Figure 7:**
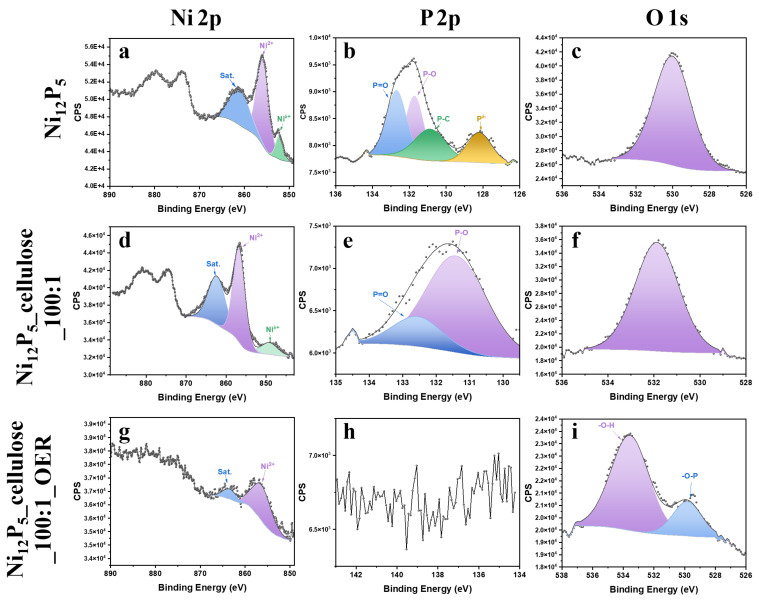
High-resolution XPS spectrum of samples: Ni_12_P_5_, Ni_12_P_5__cellulose_100:1, and Ni_12_P_5__cellulose_100:1_OER. Ni 2p core level spectra (**a**,**d**,**g**), P 2p core spectra (**b**,**e**,**h**), O 1s core spectra (**c**,**f**,**i**) of Ni_12_P_5_ sample (**top**), Ni_12_P_5__cellulose_100:1 (**middle**) and Ni_12_P_5__cellulose_100:1_OER (**bottom**).

**Figure 8:**
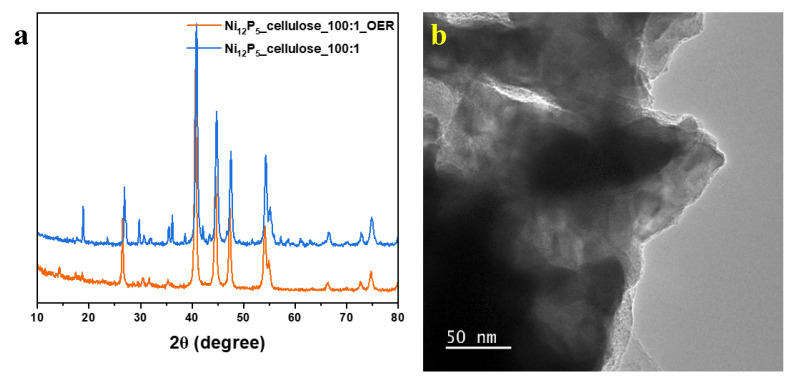
(**a**) XRD pattern of sample Ni_12_P_5__cellulose_100:1 before and after OER, and (**b**) TEM image of Ni_12_P_5__cellulose_100:1.

XPS analysis of three samples: Ni_12_P_5_, Ni_12_P_5__cellulose_100:1, and Ni_12_P_5__cellulose_100:1_OER was performed. The overall survey spectrums show signals from P, O, and Ni (see Figure S7). Additionally, in the sample of Ni_12_P_5__cellulose_100:1_OER, clear signals from the F 1s and C 1s regions are presented due to residue of Nafion^®^ and carbon foil after collecting the materials from the electrode. The XPS spectra in the Ni (2p), P (2p), and O (1s) regions for the three catalysts are shown in Figure 7. For all samples, the Ni 2p spectrum presents two main structures, resulting from the spin-orbit spinning of the p orbitals that are assigned to Ni 2p_3/2_ (850–865 eV) and Ni 2p_1/2_ (865–890 eV). Ni 2p_3/2_ of all samples presents a main peak around 856.9 eV, which is assigned Ni^2+^ oxidation states of NiO, due to air exposure, and a satellite peak located at 862.4 eV [49,50]. Moreover, both Ni_12_P_5_ and Ni_12_P_5__cellulose_100:1 exhibit a clear signal located near 852.8 eV, which is close to the Ni^0^ signal (852.6 eV), suggesting that the Ni in phosphide possesses a small partial positive charge (Ni^δ+^, 0 < δ < 2) and is assigned to nickel bonding in nickel phosphides [49,51]. The lack of this signal in the Ni_12_P_5__cellulose_100:1_OER sample suggests that during the OER process, the surface of nickel phosphides is oxidized to NiO. Moreover, the low intensity of the nickel signal for this sample indicates the migration of particles into the sample, depositing nickel oxide on the surface, which is also indicated by the lack of phosphorus signal for this sample. The analysis of P 2p spectra of Ni_12_P_5_ shows two peaks located at 128.2 eV and 131.8 eV, which can be deconvoluted to three signals located at 131.8 eV and 132.8 eV corresponding to P-O and P=O bonds, respectively. The presence of P-O and P=O bonding is due to the surface oxidation of the sample. The binding energy at 128.2 eV is lower than that of P^0^ (130.0 eV), indicating that P species have a partial negative charge (P^δ−^, 0 < δ < 1) and can be assigned to P^0^ in phosphides [51,52]. The peak in the P 2p region of Ni_12_P_5__cellulose_100:1 can be deconvoluted to two sub-peaks located at 133.4 eV and 134.6 eV, which are assigned to phosphate formation in the sample. The lack of P 2p signal for the Ni_12_P_5__cellulose_100:1_OER sample indicates restructuration of the surface of the sample, leading to the migration of P atoms to deeper layers of the materials, which was confirmed by the XRD results (see Figure 8). Also, the contamination of graphite foil and Nafion^®^ in the sample translates to signal deterioration. The peaks located near 531 eV for the samples Ni_12_P_5_, Ni_12_P_5__cellulose_100:1, and Ni_12_P_5__cellulose_100:1_OER correspond to O-P [50,52]. Moreover, for the sample Ni_12_P_5__cellulose_100:1_OER, a high-intensity signal located near 534 eV is assigned to adsorbed H_2_O or -OH groups at or within interface due to electrolyte exposure [53,54,55]. From the information provided, it can be inferred that during the OER process, nickel phosphide undergoes oxidation to form NiO. Additionally, there is migration of phosphorus atoms within the sample, and oxygen, in the form of water molecules or -OH groups, becomes adsorbed on the surface.

### 2.2. Proposed Mechanism

The exploration of the catalytic activity mechanism holds significant interest due to its potential to advance the development of more effective and sustainable energy conversion technologies. Therefore, here, we endeavor to delve into the mechanism of Ni_12_P_5__cellulose_100:1, elucidating the role of each composite component and its synergy through analysis of XRD and TEM analyses of this sample after the OER process.

To explore alterations occurring during the oxygen evolution process, several experiments were conducted to examine chemical transformations in the most favorable electrocatalyst, namely Ni_12_P_5__cellulose_100:1. The specimen after the OER was obtained through sonication of the graphitic foil with electrocatalysts after the LSV measurement. Subsequently, it was purified from Nafion by rinsing it with isopropanol and water. The dried specimen underwent vacuum drying at 80 °C under 35 mbar for 24 h. This resulting sample is denoted as Ni_12_P_5__cellulose_100:1_OER. Figure 8 presents the XRD patterns of the Ni_12_P_5__cellulose_100:1 sample before and after the OER. The XRD patterns are not changed, showing the same signals, suggesting that there are no changes in the material composition during the process. This conclusion was also confirmed by TEM analysis, which shows Ni_12_P_5_ particles characteristic of the material analyzed before the OER (Figure 3). This proves the stability and potential of this composition and indicates that the sample does not undergo undesired intermediate reactions and unwanted products, which proves its stable behavior. 

Based on the latest advancements, nearly all electrocatalysts relying on transition metal phosphides (such as Co-P, Fe-P, and Ni-P) demonstrate similar mechanisms for the OER reaction in alkaline conditions, with varying rate-determining steps in such an environment. Currently, the most acknowledged OER pathways in alkaline media are illustrated as

(1) $$M+{OH}^{-}\to {M-OH}_{ads}+e^{-}$$

(2) $${M-OH}_{ads}+{OH}^{-}\to {M-O}_{ads}+H_{2}O+e^{-}$$

(3) $${M-O}_{ads}+{M-O}_{ads}\to 2M+O_{2}$$

(4) $${or\;M-O}_{ads}+{OH}^{-}\to M-{OOH}_{ads}+e^{-}$$

(5) $$M-{OOH}_{ads}+{OH}^{-}\to M+O_{2}+H_{2}O+e^{-}$$

where M stands for the active site of the surface of the electrocatalyst, and “ads” is the adsorption species on the surface of the catalyst [35,56]. Fundamentally, all proposed mechanisms initiate with hydroxide coordination to the active site (1), followed by two primary pathways: the direct linking of two M-O_ads_ intermediates ((1) → (2) → (3)) and steps (1) → (2) → (4) → (5), wherein M-OOH_ads_ is formed and subsequently combines with OH^-^ to generate O_2_. Across all metal phosphides, the OER reaction in alkaline media predominantly follows the second pathway. This preference is attributed to the consistent observation that the thermodynamic barrier of reaction (3) is consistently higher than that of (4) and (5).

**Figure 9:**
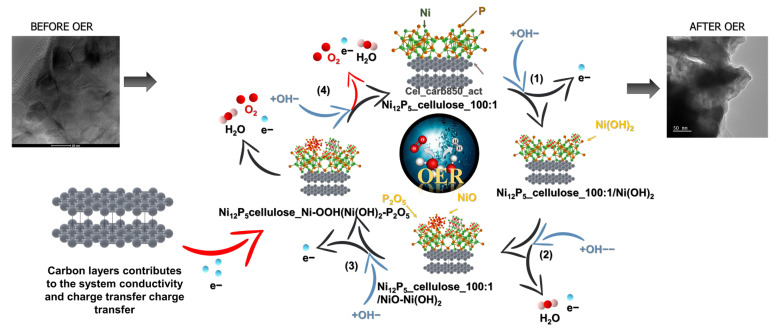
Graphical representation of reaction mechanism.

Therefore, it is believed that, at first, the surface of Ni_12_P_5_ is oxidized upon the applied external voltage. Then, the water molecule is adsorbed on the surface of Ni_12_P_5__cellulose_100:1(NiO_2_). The hydroxide ion (OH^−^) from the electrolyte react with the water molecule adsorbed on the catalyst’s surface, causing the formation of the Ni-O_ads_ form, which further reacts with the hydroxide ion from the electrolyte resulting in Ni-OOH_ads_ formation, subsequently combined with OH^−^ to produce O_2_. Nevertheless, throughout the process, the phosphorus atoms carry a higher positive charge compared to nickel, facilitating the easier adsorption of OH^−^ in the initial reaction. This characteristic contributes to an overall enhancement in the efficiency of the OER process [57]. By combining Ni_12_P_5_ with other elements, Ni atoms receive a partial electron from another element, which may result in a decrease in the adsorption energies of reaction intermediates [58]. On the other hand, the presence of carbon contributes to the OER kinetics by providing more electrical contacts between active species and electrolytes for faster charge transfer reactions [59]. The schematic representation of this processes is shown in Figure 9. In summary, by combining the positive influence of carbon with Ni_12_P_5_, we have developed the optimal mass ratio which allowed us to obtain an effective catalyst due to the synergy of the positive features of these compounds.

## 3. Experimental Section

### 3.1. Materials and Chemicals

Cellulose fibers were derived by Arctic Paper Kostrzyn SA, Kostrzyn nad Odrą, Poland. Nickel (II) sulfate hexahydrate (NiSO_4_·6H_2_O, CAS: 10101-97-0) and red phosphorous (P, CAS: 7723-14-0) were purchased from Sigma Aldrich, St. Louis, MO, USA, and were used without further modifications. Potassium hydroxide (≥95%, KOH) and ethylene glycol (EG) were delivered from Chempur (Piekary Śląskie, Poland).

#### 3.1.1. Preparation of Cellulose Fibers

To enhance the carbonization efficiency of cellulose fibers, the initial step involved ball milling of eucalyptus-derived cellulose fibers to produce a fine powder. Before ball milling, the cellulose, sourced from a carton board, was divided into smaller sections through grinding. Subsequently, the processed material was placed in an agate jar along with 30 agate balls, each with a 10 mm diameter. The ball milling program was configured for 2 cycles, with each cycle lasting 30 min at a speed of 500 rpm and a 10 min pause between cycles.

#### 3.1.2. Carbonization of Cellulose 

The cellulose powder was loaded into a ceramic boat and introduced into a vertical furnace under a nitrogen atmosphere. The carbonization process was carried out at different temperatures, specifically 650 °C, 750 °C, 850 °C, and 950 °C. The samples were maintained at these temperatures for 2 h before undergoing natural cooling to room temperature. The samples were labeled as cel_carb_X, with X representing the respective process temperature (650 °C, 750 °C, 850 °C, and 950 °C).

#### 3.1.3. Activation of Carbonized Cellulose

To enhance the specific surface area and micropore volume, an activation process was initiated. Initially, carbonized cellulose and KOH in a 1:4 mass ratio were finely ground in a mortar to achieve a uniform powder. Subsequently, the powder was placed in a ceramic boat and introduced into a vertical furnace, where it was heated to 800 °C for a 2 h reaction time. Next, the furnace was naturally cooled to room temperature, and the resulting sample was transferred to a beaker containing 200 mL of 1 M HCl solution and stirred for 12 h. Afterward, the solution underwent filtration, multiple washing with water to achieve a neutral pH value, and drying in an oven at 80 °C. These samples were labeled as cel_carb_X_act, with X representing the temperature of the process (650 °C, 750 °C, 850 °C, and 950 °C).

#### 3.1.4. Synthesis of Nickel Phosphide (Ni_12_P_5_)

To obtain nickel phosphide nanoparticles, NiSO_4_·6H_2_O and red phosphorous were combined in a molar ratio of Ni:P equal to 3:2, along with a solvent mixture (V_EG_/V_H2O_ = 1:4), using a magnetic stirrer. The resulting clear solution was then transferred into a 100 mL Teflon-lined autoclave, sealed, and heated to 180 °C for 12 h. After that, the product was cooled down to room temperature. Subsequently, the product was filtered and washed with ethanol and distilled water to remove excess of red phosphorous and other by-products of the reaction. After that, the prepared Ni_12_P_5_ was dried at 100 °C. 

#### 3.1.5. Synthesis of Cellulose and Nickel Phosphide Composite (Ni_12_P_5__Cellulose)

To obtain nickel phosphide nanoparticles deposited on cellulose nanosheets, the desired amount of NiSO_4_·6H_2_O and red phosphorous with a Ni:P molar ratio of 3:2 and 500 mg of cel_carb_850_act were combined and were mixed using a magnetic stirrer in the solvent mixture (V_EG_/V_H2O_ = 1:4). The resulting solution was then transferred into a 100 mL Teflon-lined autoclave, sealed and heated to 180 °C, and maintained at this temperature for 12 h. After that, the product was naturally cooled down to room temperature. Subsequently, the product was filtered and washed with ethanol and distilled water to remove excess of red phosphorous and other by-products of the reaction. Finally, the prepared Ni_12_P_5_ was dried at 100 °C. The samples were labeled as Ni_12_P_5__cel_X, with X representing the mass ratio of Ni_12_P_5_ to cel_carb_850_act (100:1, 10:1, 1:1, 10:1, 100:1).

### 3.2. Characterization Methods

Scanning electron microscopy (SEM, TescanVega3, TESCAN, Brno, Czech Republic) with an accelerating voltage of 30 kV was employed to observe the morphology of samples. High-resolution transmission electron microscopy (HR-TEM, Thermo Fisher Scientific, Waltham, MA, USA) and imaging were performed with the Spectra 200 microscope at an accelerating voltage of 300 kV. The chemical composition of the studied materials was established using X-ray Powder Diffraction (Aeris, Malvern Panalytical, UK) using CuKα radiation. The adsorption/desorption isotherms of N_2_ in liquid nitrogen temperature (−196 °C) were used to determine the specific surface area using the Brunauer–Emmett–Teller method (BET—Micrometric ASAP 2460, Norcross, GA, USA). Raman spectra were collected using a Renishaw (laser wavelength = 785 nm). SDT Q600 thermogravimetric analyzer (TGA, TA Instruments, New Castle, DE, USA) instrument was used to perform thermogravimetric analysis under air atmosphere with a heating rate of 10 °C/min. Atomic Force Microscopy (AFM MultiMode 8, Bruker, Billerica, MA, USA) provided information about the thickness and lattice size of the exfoliated materials. The chemical composition and relative atomic percentages on the surface of the samples were studied by X-ray Photoelectron Spectroscopy (XPS). The measurements were conducted using MgKα (hν = 1253.6 eV) radiation in a PREVAC (Rogów, Poland) system equipped with a Scienta SES 2002 (Uppsala, Sweden) electron energy analyzer operating with constant transmission energy (Ep = 50 eV). The analysis chamber was evacuated to a pressure below 5 × 10^−9^ mbar. 

### 3.3. Electrochemical Measurements

The electrochemical tests were performed by BioLogic VMP-3 (Seyssinet-Pariset France) potentiostat station in a three-electrode system with a constant temperature control bath at 25 °C ± 0.005 °C by Hubner KISS 6 (Kassel, Germany) thermostat. Mercury oxide electrode Hg|HgO (MOE) (ALS Japan, Tokyo, Japan) as the reference electrode and platinum wire (surface area~3.6 cm^2^) as the counter electrode were used. The working electrode was a 10 × 10 mm, 125-µm-thick graphite foil (99.8%, GoodFellow, Huntingdon, UK). In total, 10 mg of active material was dispersed in 1 mL solution and drop-casted on graphitic foil, and then dried for 12 h in the air. All measurements were performed in the alkaline electrolyte (1 M KOH). All measurements were acquired according to the Reversible Hydrogen Electrode (RHE) potential calculated by the equation:

$$E_{RHE}=E_{WE}+E^{0}+pH\times 0.059$$

where *E_RHE_*, *E_WE_*, and *E*^0^ correspond to the potential of the reversible hydrogen electrode, working electrode (WE) potential, and standard potential of the reference electrode (*E*^0^*_MOE_* = 0.128 V), respectively. 

Electrochemical impedance spectroscopy (EIS) measurements were conducted at the current density of 5 mA·cm^−2^, with a potential amplitude of 10 mV, in the frequency range of 100 mHz–200 kHz.

## 4. Conclusions

In summary, a practical and facile pathway for highly porous carbon flakes derived from cellulose fibers as a platform for nickel phosphide nanoparticles has been developed. The optimized route (850 °C) allowed us to provide defected carbon flakes with a very high specific surface area after the activation process (SSA_cel_carb_850_act_ = 3164 m^2^/g). Next, the carbon matrix was used to design the most efficient electrocatalyst via combination with different ratios of Ni_12_P_5_ to cellulose. As prepared electrocatalysts were tested towards the OER. It was clearly revealed that the most favorable kinetics and robustness in the OER process were evaluated for the composite of Ni_12_P_5__cellulose_100:1, proving its practical potential. Additionally, the analysis of the electrocatalyst material after the OER allowed us to conclude that the reaction does not involve any intermediates and side-reactions, which is additionally beneficial in robust systems. Therefore, we propose a facile route to fabricate a cellulose-based composite with promoted electroactivity and durability. It is believed that this research can be extended into other nickel phosphides or bi-functional nickel phosphides being anchored on wasted cellulose, providing more sustainable technology.

## Figures and Tables

**Table 1:** I_D_/I_G_ ratio values, BET specific surface area, micropore volume, and micropore area of cellulose after the carbonization with different temperatures.

Sample	I_D_/I_G_	Specific Surface Area (m^2^/g)	Micropore Volume (cm^3^/g)	Micropore Area (m^2^/g)
cel_carb_650_act	1.41	1483	0.46	1100
cel_carb_750_act	1.56	1183	0.38	904
cel_carb_850_act	1.39	3164	2.25	2543
cel_carb_950_act	1.53	496	0.29	548

**Table 2:** Electrochemical properties of RuO_2_, Ni_12_P_5_, and Ni_12_P_5__cellulose composites during OER in 1.0 M KOH.

	R1	R2	Q2
Ni_12_P_5_	0.988	3.47	0.02771
Ni_12_P_5__cellulose_1:100	1.470	4.473	0.00993
Ni_12_P_5__cellulose_1:10	1.128	3.542	0.001172
Ni_12_P_5__cellulose_1:1	1.400	3.770	0.01152
Ni_12_P_5__cellulose_10:1	1.002	3.758	0.0090
Ni_12_P_5__cellulose_100:1	1	2.701	0.0031
RuO_2_	1.131	3.927	

## Data Availability

Publicly available datasets were analyzed in this study. The data are available on the Bridge of Knowledge repository be found here: [https://mostwiedzy.pl/en] under the name “Highly Porous Carbon Flakes Derived from Cellulose and Nickel Phosphide Heterostructure towards Efficient” and the data that support the findings of this study are available from the author, K.M. (klaudia.maslana@zut.edu.pl), upon request.

## Supplementary Materials

The following supporting information can be downloaded at: https://www.mdpi.com/article/10.3390/molecules29020352/s1, Figure S1: SEM images of pure cellulose fibers (a, b) and cellulose fibers after the ball-milling process (c, d).; Figure S2: TEM images of (a) cel_carb_650, (b) cel_carb_750, (c) cel_carb_850, and (d) cel_carb_950.; Figure S3: (a) Raman spectra, (b) XRD patterns, and (c) TGA patterns of cellulose and carbonized cellulose with different temperatures.; Figure S4: TEM images of (a) Ni_12_P_5__cel_100:1, (b) Ni_12_P_5__cel_10:1, (c) Ni_12_P_5__cel_1:1, (d) Ni_12_P_5__cel_1:10, and (e) Ni_12_P_5__cel_1:100; Figure S5: AFM image of Ni_12_P_5__cellulose_100:1 and corresponding height profile; Figure S6: XRD patterns of Ni_12_P_5_ and composites of Ni_12_P_5__cellulose with different ratios..; Figure S7: XPS survey scans of Ni_12_P_5_, Ni_12_P_5__cellulose_100:1, and Ni_12_P_5__cellulose_100:1_OER.; Table S1: ID/IG ratio values and BET specific surface area of cellulose after the carbonization with different temperatures..
