# Supplementary material for: Highly Porous Carbon Flakes Derived from Cellulose and Nickel Phosphide Heterostructure towards Efficient Electrocatalysis of Oxygen Evolution Reaction
Source: Molecules. 2024 Jan 10;29(2):352. doi: 10.3390/molecules29020352 (PMC10819855; doi:10.3390/molecules29020352)
Supplement: Supplementary file 1 [file molecules-29-00352-s001.zip › molecules-2775984-supplementary.pdf]

Supplementary Information

Highly Porous Carbon Flakes Derived from Cellulose and Nickel Phosphide Heterostructure towards Efficient Electrocatalysis of Oxygen Evolution Reaction

Ewa Mijowska [1,2,*], Karolina Pietrusewicz [1] and Klaudia Maślana [1,2]

[1] Department of Nanomaterials Physicochemistry, Faculty of Chemical Technology and Engineering, West Pomeranian University of Technology, Piastow Ave. 45, 70-311 Szczecin, Poland
[2] Center for Advanced Materials and Manufacturing Process Engineering (CAMMPE), West Pomeranian University of Technology, 70-310 Szczecin, Poland
* Correspondence: emijowska@zut.edu.pl

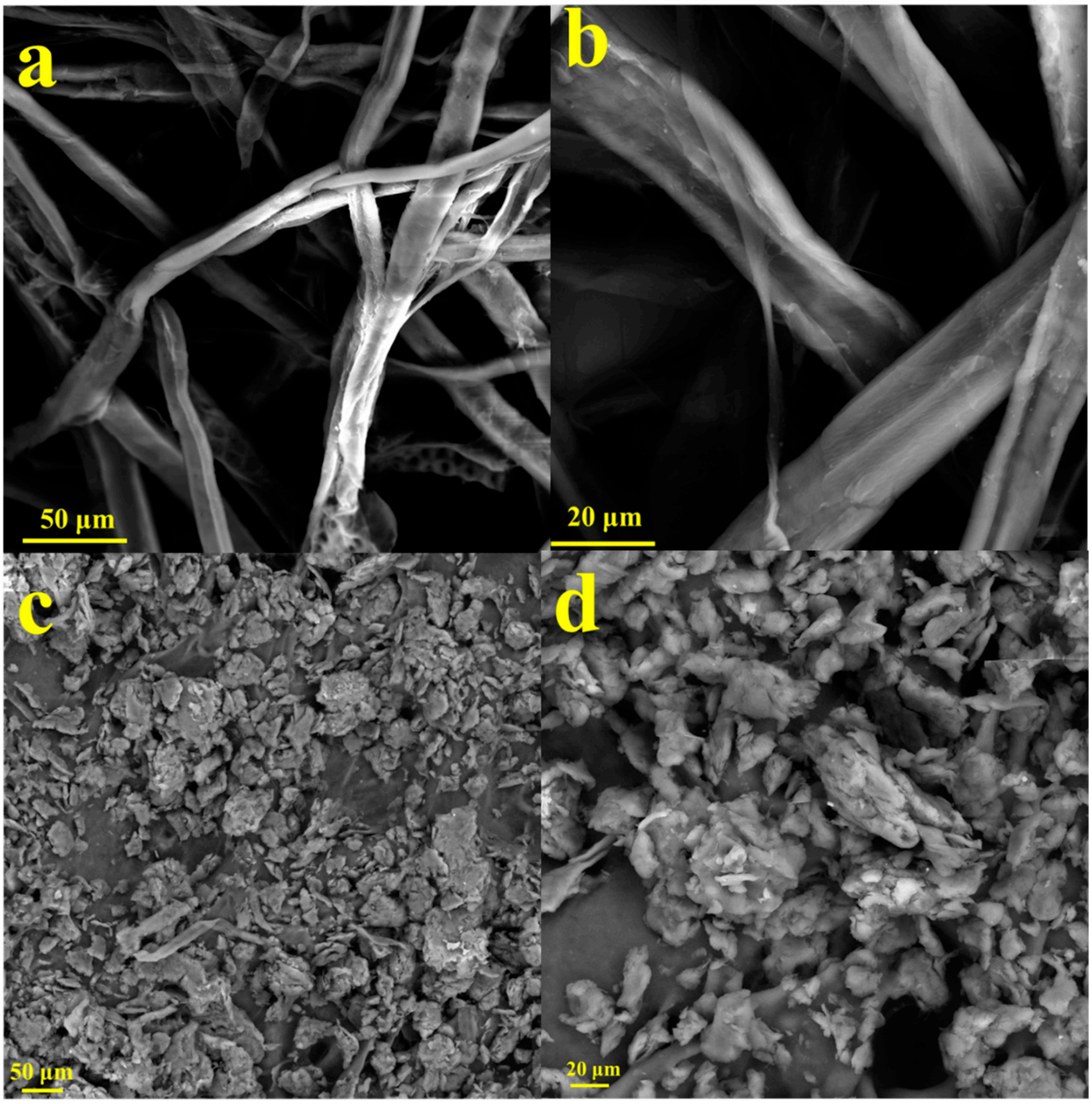

Figure S1. SEM images of pure cellulose fibers (a,b) and cellulose fibers after the ball-milling process (c,d).

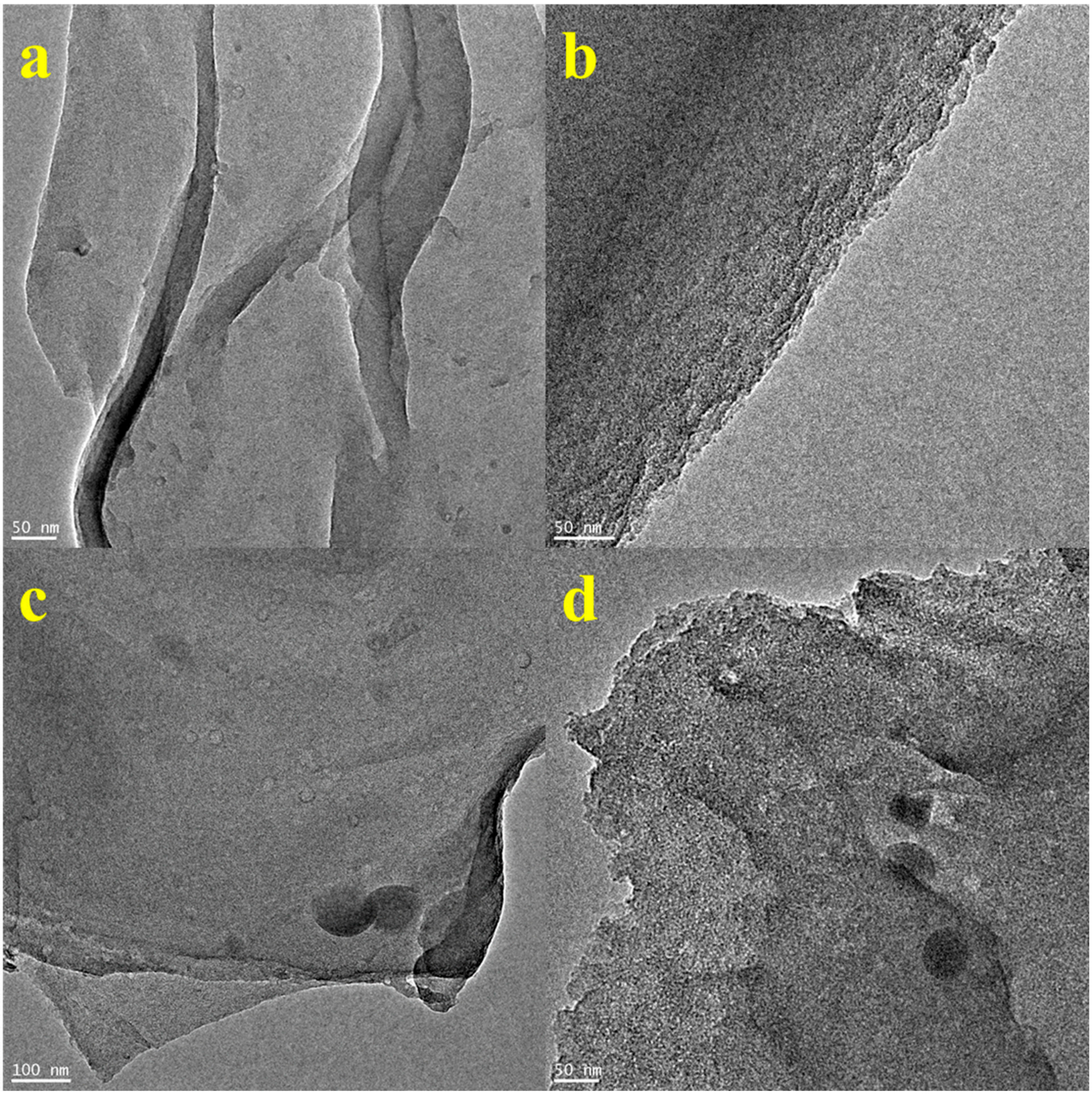

Figure S2. TEM images of (a) cel_carb_650, (b) cel_carb_750, (c) cel_carb_850, and (d) cel_carb_950.

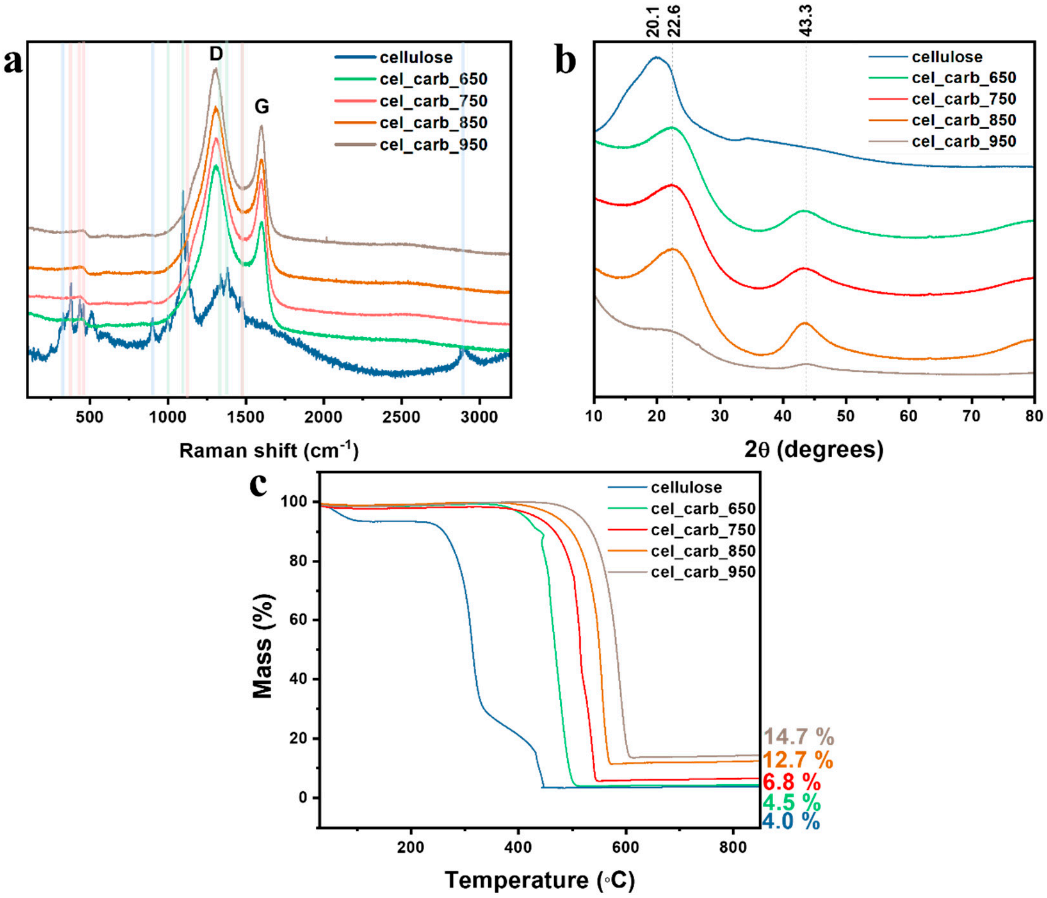

Figure S3. (a) Raman spectra, (b) XRD patterns, and (c) TGA patterns of cellulose and carbonized cellulose with different temperatures.

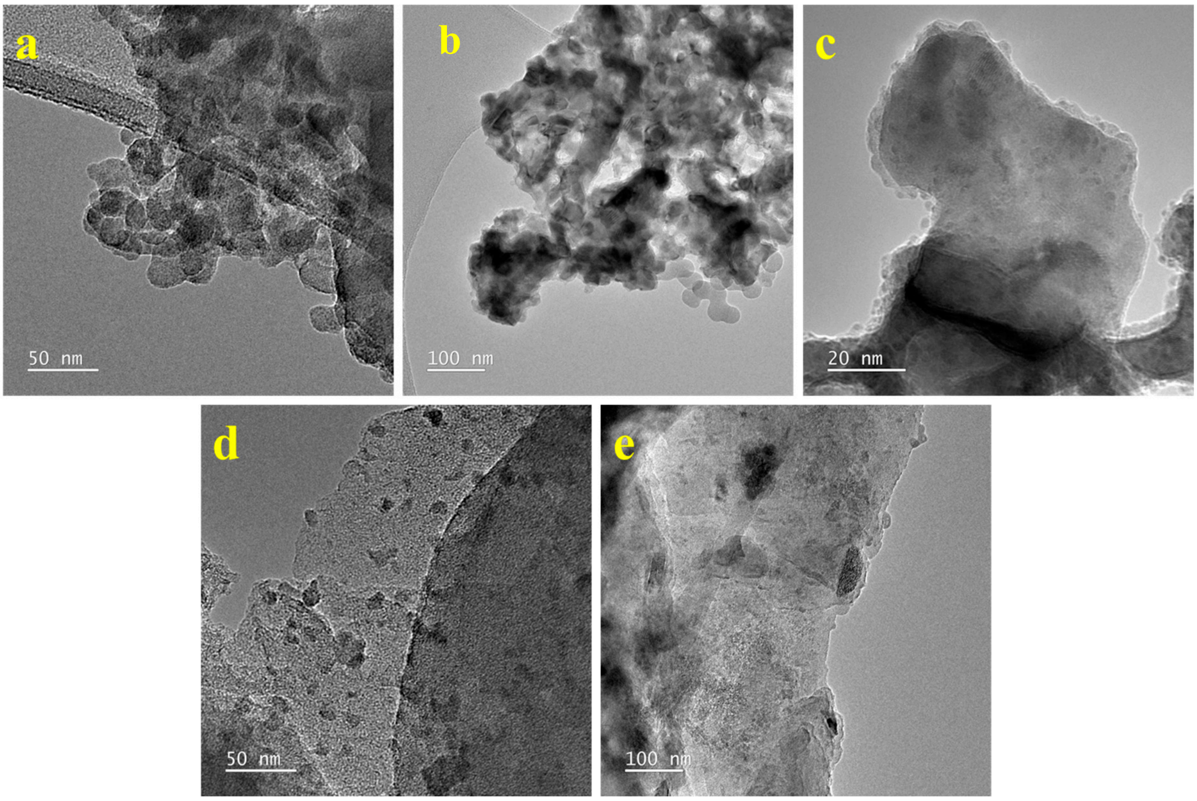

Figure S4. TEM images of (a) $Ni_{12}P_5$_cel_100:1, (b) $Ni_{12}P_5$_cel_10:1, (c) $Ni_{12}P_5$_cel_1:1, (d) $Ni_{12}P_5$_cel_1:10, and (e) $Ni_{12}P_5$_cel_1:100.

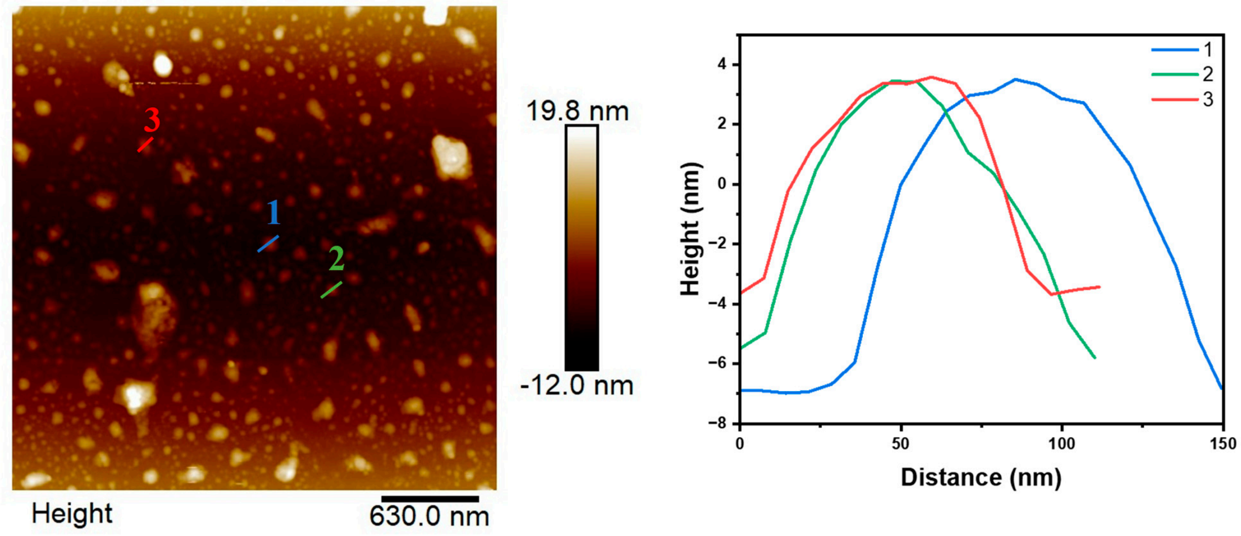

Figure S5. AFM image of $Ni_{12}P_5$_cellulose_100:1 and corresponding height profile.

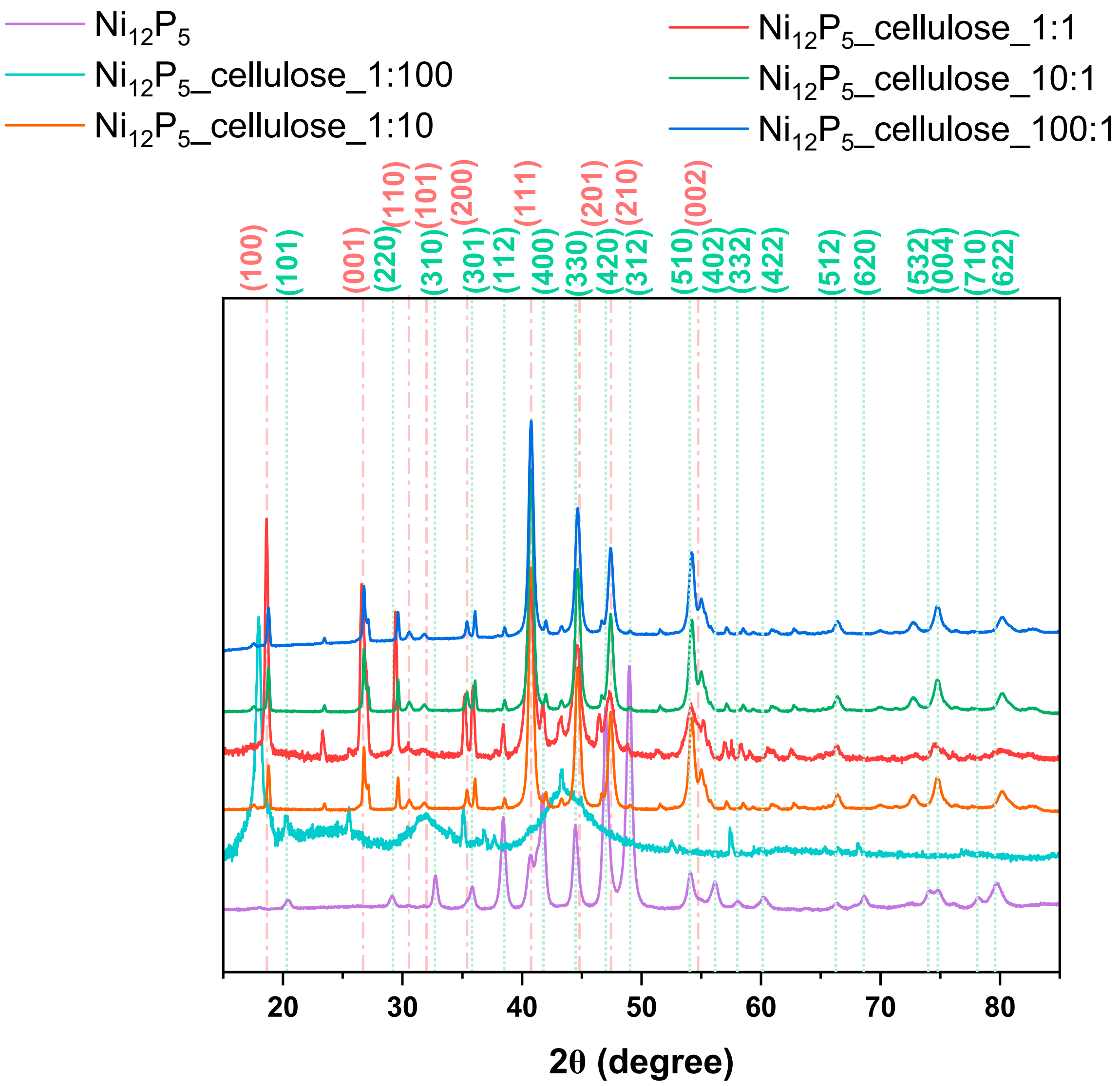

Figure S6. XRD patterns of $Ni_{12}P_5$ and composites of $Ni_{12}P_5$_cellulose with different ratios.

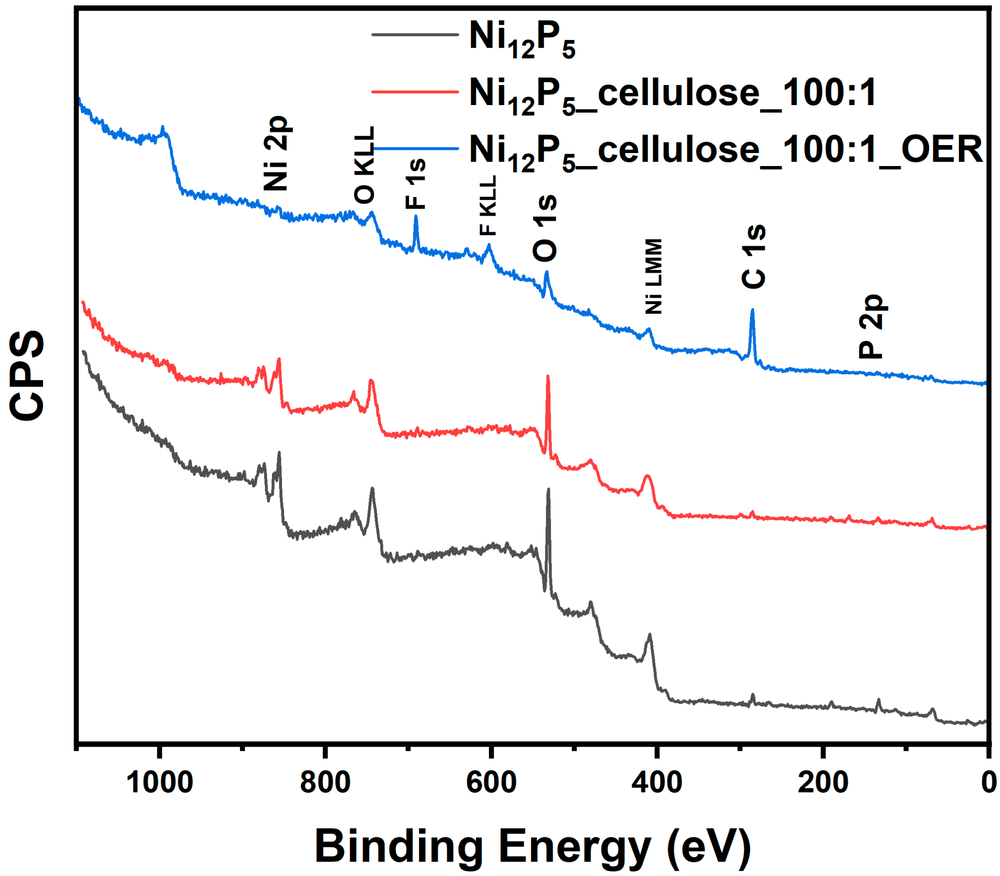

Figure S7. XPS survey scnas of $Ni_{12}P_5$, $Ni_{12}P_5$_cellulose_100:1, and $Ni_{12}P_5$_cellulose_100:1_OER.

Table S1. I_D/I_G ratio values and BET specific surface area of cellulose after the carbonization with different temperatures.

Sample	**I_D/I_G ratio**	**Specific surface area (m^2/g)**
cel_carb_650	1.229	301 m^2/g
cel_carb_750	1.309	342 m^2/g
cel_carb_850	1.192	374 m^2/g
cel_carb_950	1.484	589 m^2/g
